# High rate of invasive fungal infections during early cycles of azacitidine for patients with acute myeloid leukemia

**DOI:** 10.3389/fcimb.2022.1012334

**Published:** 2022-11-30

**Authors:** Sing-Ting Wang, Chia-Huei Chou, Tzu-Ting Chen, Ching-Chan Lin, Li-Yuan Bai, Shih-Peng Yeh, Mao-Wang Ho, Ming-Yu Lien

**Affiliations:** ^1^ Division of Hematology and Oncology, Department of Internal Medicine, China Medical University Hospital, Taichung, Taiwan; ^2^ Division of Infection Disease, Department of Internal Medicine, China Medical University Hospital, Taichung, Taiwan; ^3^ Department of Internal Medicine, Graduate Institute of Clinical Medicine, China Medical University, Taichung, Taiwan; ^4^ Graduate Institute of Basic Medical Science, China Medical University, Taichung, Taiwan

**Keywords:** azacitidine, acute myeloid leukemia, invasive fungal infection, venetoclax and azacitidine, Taiwan hospital

## Abstract

**Background:**

Acute myeloid leukemia (AML) is a form of cancer that is characterized by infiltration of the bone marrow, blood, and other tissues by proliferative, clonal, abnormally differentiated, and occasionally poorly differentiated cells of the hematopoietic system. Patients with acute myeloid leukemia (AML) receiving azacitidine (AZA) alone or in combination with venetoclax (VEN-AZA) are at increased risk for invasive fungal infections (IFIs). We compared the incidence and risk of IFI during these treatment regimens in a single Taiwan hospital.

**Materials and methods:**

A total of 61 patients with AML received at least one course of AZA in the hematology ward of China Medical University Hospital (Taichung, Taiwan) between September 2012 and June 2020. Thirty-eight patients (62.3%) received AZA monotherapy; 23 (37.7%) received VEN-AZA.

**Results:**

Incidence rates of probable and proven IFI were 18% and 1.6%, respectively, during AZA treatment. One proven case of *Fusarium* spp. infection was isolated by skin and soft tissue culture. Most (75%) IFI cases occurred during the first cycle of AZA therapy. Half of all IFI cases occurred in patients with prolonged neutropenia. The risk of IFI was significantly higher for the European LeukemiaNet (ELN) nonfavorable-risk group (intermediate- and adverse-risk group) versus the ELN favorable-risk group and for patients with prolonged neutropenia versus those without (*P*<0.05 for both comparisons). In this study, median OS did not differ significantly between patients with and without IFIs during AZA-containing regimens (14.6 months vs 13.7 months; *P*=0.59).

**Conclusion:**

The incidence of IFI was high in this AML cohort treated with AZA-containing regiments in Taiwan. The majority of IFI cases occurred during the early cycles of AZA (cycles 1–2). Prospective studies are needed to determine the optimal choice of antifungal prophylaxis agent during VEN-AZA therapy for AML.

## Introduction

Acute myeloid leukemia (AML) is a heterogenous hematologic malignancy characterized by infiltration of the bone marrow, blood, and other tissues by proliferative, clonal, abnormally differentiated, and occasionally poorly differentiated cells of the hematopoietic system (Döhner et al., 2015). AML can arise in patients with a consequence of prior therapy such as exposure to topoisomerase II, alkylating agents or radiation or an underlying hematological disorder (Döhner et al., 2015). However, most of them appears as a *de novo* malignancy in previously healthy individuals. Regardless of the etiology, the pathogenesis of AML involves the clonal expansion and abnormal proliferation of myeloid stem cells (De Kouchkovsky and Abdul-Hay, 2016). Invasive fungal infections (IFIs) are an important contributor to mortality during induction chemotherapy for acute myeloid leukemia (AML) (Othus et al., 2014; Lin et al., 2018). We recently reported a high IFI incidence rate of 20% for proven or probable IFI in a Taiwanese cohort of newly-diagnosed patients with AML receiving induction chemotherapy; the IFI rate increased to 33% when combined with possible cases (Lien et al., 2018). The occurrence of IFIs during first induction chemotherapy has a significant negative impact on AML survival (Candoni et al., 2020). Antifungal prophylaxis for the prevention of IFI is endorsed by international guidelines, such as those issued by the European Conference on Infections in Leukemia (ECIL), which recommends posaconazole as the drug of choice for primary antifungal prophylaxis in patients with AML receiving induction chemotherapy (Kung et al., 2018; Maertens et al., 2018), although antifungal prophylaxis does not necessarily mean an insignificant level of mortality amongst older patients undergoing intensive consolidation therapy for AML (Del Principe et al., 2019).

The hypomethylating agents (HMAs) azacitidine (AZA) and decitabine (DAC) are the treatment of choice for patients with AML unable to tolerate intensive chemotherapy, particularly those aged ≥65 years (Xu et al., 2021). Compared with conventional care regimens, AZA has been shown to significantly prolong overall survival (OS) in patients aged ≥65 years with newly-diagnosed AML and >30% bone marrow blasts (Dombret et al., 2015). Venetoclax (an oral inhibitor of B-cell lymphoma 2, BCL-2) has demonstrated synergy and good tolerability in combination with either AZA or DAC in treatment-naïve patients aged ≥65 years with AML deemed unsuitable for chemotherapy (DiNardo et al., 2019). In even older-aged patients (≥75 years), a phase 3 randomized, placebo-controlled trial revealed that AZA plus venetoclax (VEN) was superior to AZA monotherapy, with higher incidence rates of both complete remission (CR; 36.7% vs 17.9%, respectively; *P*<0.001) or CR with incomplete hematologic recovery (66.4% vs 28.3%; *P*<0.001) (DiNardo et al., 2020). Increasingly, treatment regimens favor HMAs alone or in combination with venetoclax (VEN-HMA) for elderly, treatment-naïve patients with AML who cannot undergo intensive chemotherapy, or for cases of relapsed or refractory disease.

HMAs and especially VEN-HMAs regimens have been associated with a significantly higher risk of neutropenia and thrombocytopenia, increasing the risk of fungal infection in patients (Gao et al., 2018). In previous published studies, the IFI percentages range from between 1.6% and 12.5% in acute myeloid leukemia/myelodysplastic syndrome (AML/MDS) populations administered HMA-containing regiments (Falantes et al., 2014; Pomares et al., 2016; Ali et al., 2017; Trubiano et al., 2017; Mądry et al., 2019; Kim et al., 2020). In one study involving 145 treatment-naïve, elderly patients with AML administered VEN-HMAs, grade 3/4 adverse events included febrile neutropenia (43%) and neutropenia (17%), and a low rate of fungal infections (8%) that was attributed to the fact that 46% of these patients received non-azole antifungal prophylaxis (Aldoss et al., 2019). Drawbacks of these observation studies include the fact that not only do they differ in IFI diagnostic criteria and their use of antifungal prophylaxis drugs, but they also targeted patients with mixed AML and MDS. Little is known about the value of antifungal prophylaxis in AML patients receiving HMAs or VEN-HMAs. Moreover, consistent recommendations are lacking as to appropriate antifungal prophylaxis in this patient population.

We sought to characterize the epidemiology, risk factors and outcomes of IFIs in patients with AML administered AZA-only or VEN-AZA regimens in Taiwan, where the typically humid, moist environment encourages fungal growth. We also examined the incidence of IFI and assessed the potential need for antifungal prophylaxis with both treatment regimens, as well as the appropriateness and efficacy of what the study participants received.

## Materials and methods

### Patients

**Figure 1:**
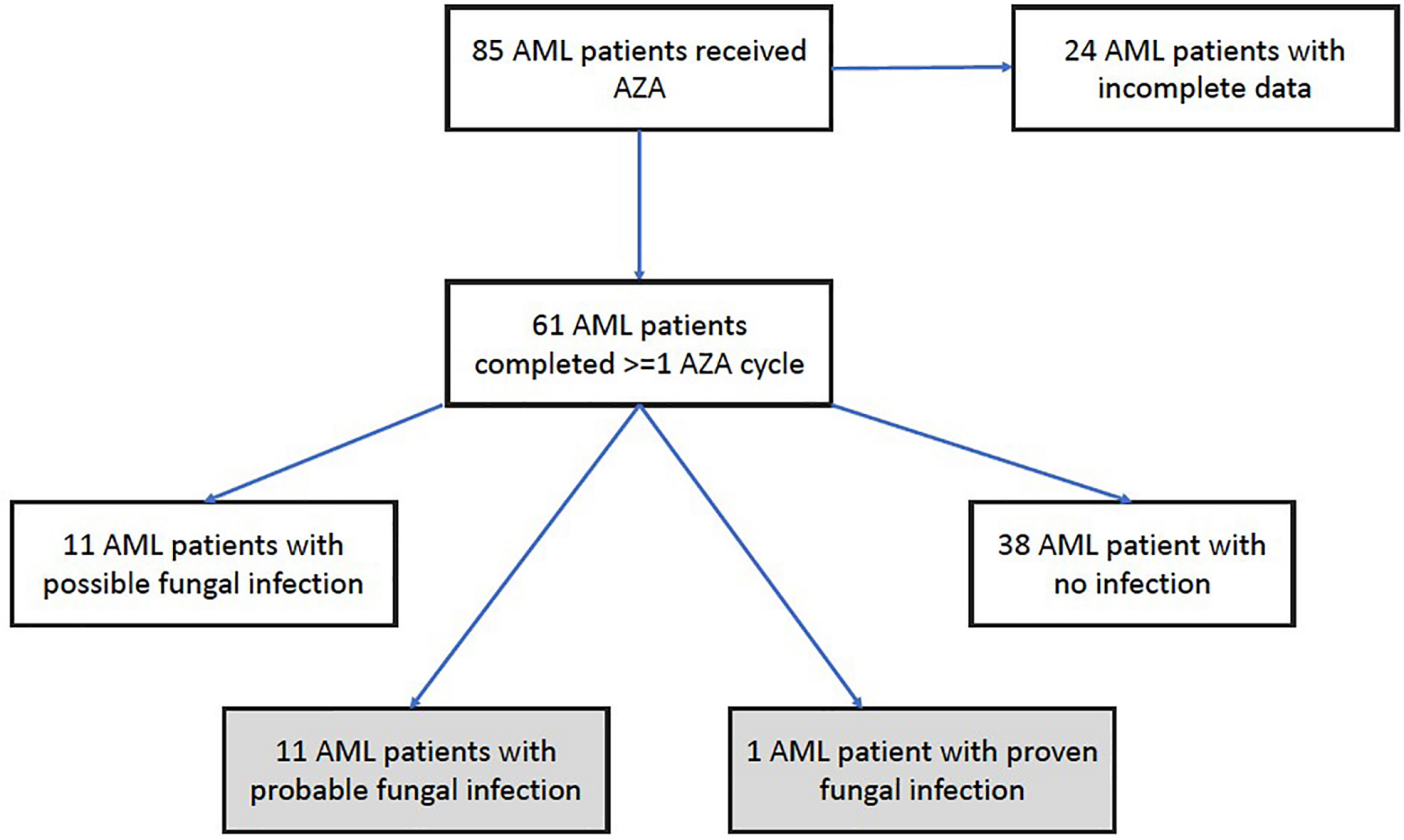
Patients distribution in the study. A total of 61 AML participants were recruited into the study. 11 cases of probable fungal infection. 1 patient of proven fungal infection.

We obtained data on IFI incidence rates involving all adult patients (aged >18 years) treated with at least 1 courses of AZA-containing regimens for AML in the hematology ward of China Medical University Hospital (Taichung, Taiwan) between September 2012 and June 2020. All data were charted in the electronic medical records and during clinical follow-up visits (**Figure 1**). AZA was given at the standard recommended dose of 75mg/m^2^/day for 7 days every 4 weeks, without routine primary antifungal prophylaxis (Taplitz et al., 2018). Any patients administered AZA with a previously documented fungal infection receiving secondary prophylaxis or an adapted antifungal therapy regimen were excluded from the analysis. For the VEN-AZA regimen, the venetoclax dosage was 100 mg once daily to account for expected increases in venetoclax plasma concentrations caused by posaconazole (300 mg once daily). AML cytogenetic risk stratification and AML-related treatment responses were evaluated according to the European LeukemiaNet (ELN) recommendations issued in 2017 (Döhner et al., 2017). ELN risk classification (favorable, intermediate, or adverse) was determined by conventional cytogenetics, in combination with next-generation sequencing (NGS) data when available. A neutropenic episode was defined as an absolute neutrophil count of <500 cells/µL and prolonged neutropenia was defined as an absolute neutrophil count (ANC) ≤500 cells/µL lasting >7 days. Thrombocytopenia was defined as a platelet count of <25,000/µL. We recorded all neutropenic and thrombocytopenic episodes that occurred 30 days before or after the diagnosis of IFI. Bone marrow studies assessing treatment responses in AML patients were usually conducted 2–6 months after AZA monotherapy. In the VEN-AZA cohort, bone marrow studies were usually conducted 1–3 months after treatment. We determined the best response after treatment for all patients, as per the ELN recommendations in 2017.

All study procedures followed the ethical standards of the Research Ethics Committee of China Medical University Hospital and the ethical principles issued by the Declaration of Helsinki covering medical research involving humans. The requirement for informed consent was waived for this study by the Ethics committee because the data were analyzed anonymously (Approval No.: CMUH109-REC1-168).

### Invasive fungal infections

The diagnostic workup included blood culture, chest radiograph at fever onset, galactomannan (GM) testing and chest computed tomography (CT) imaging at 7 days after the onset of fever. Other examinations (e.g., abdominal ultrasound scan, sinus or brain CT, skin and soft tissue biopsy, bronchoalveolar lavage) were performed if needed. The Platelia™ Aspergillus galactomannan antigen sandwich enzyme immunoassay (GM-EIA, Bio-RAD Laboratories) was used for prospective surveillance of invasive aspergillosis (IA) at a GM cut-point of >0.5. In this study, serum Aspergillus GM tests were considered positive if two consecutive GM-EIA values of >0.5 were obtained within 1–2-week intervals. Cases of possible, probable, and proven IFI cases satisfied by the European Organization for Research and Treatment of Cancer/Invasive Fungal Infections Cooperative Group and the National Institute of Allergy and Infectious Diseases Mycoses Study Group (EORTC/MSG) 2008 criteria. According to EORTC/MSG, proven invasive fungal infection (IFI) required only that a fungus be detected by culture of a specimen of tissue taken from a site of disease or histological analysis. Probable IFI require that a host factor, mycological evidence, and clinical features be present. And possible IFI was defined to patients with the appropriate host factors and with sufficient clinical evidence consistent with IFI but for which there was no mycological support (De Pauw et al., 2008). The choice of antifungal prophylaxis was selected by the treating physician, according to individual clinical condition. Patients with persistent fever and neutropenia were managed under a diagnostic-driven approach for antifungal therapy.

### Statistical analysis

Continuous data are presented as mean values and standard deviations, categorical data are described as frequencies and percentages. Univariate and multivariate analyses identified categorical factors associated with IFIs, estimated by Cox proportional hazards regression modeling. Each prognostic factor that was associated with a significant *p*-value of <0.05 was included in multivariate analysis. We performed another sensitivity analysis to examine the impact of “possible IFIs”. Median survival times and overall survival (OS) curves were analyzed by the Kaplan-Meier method and between-group differences in survival rates were compared using the log-rank test for patients with IFIs and those without IFIs. IFI-related mortality was defined as death with continuing signs of IFI or death occurring within 30 days after IFI diagnosis. The result was independently confirmed by two clinicians. All statistical analyses were performed using the SAS for Windows software platform version 9.4 (SAS Institute Inc., NC, USA).

## Results

### Patient characteristics

During the study period from January 2012 through June 2020, 85 patients who received AZA treatment for AML was screened; 24 had received fewer than 2 cycles of AZA treatment so did not meet inclusion criteria. They had very poor outcomes and short follow-up periods. Sixty-one adult patients with AML satisfied the inclusion criteria and had no pre-existing IFIs; their clinicopathological characteristics are represented in **Table 1**. Thirty-eight patients (62.3%) received AZA monotherapy and 23(37.7%) received VEN-AZA. Forty-eight patients (80.3%) received AZA as frontline treatment; 12 (19.7%) received AZA for relapsed or refractory disease after previous intensive chemotherapy for AML. The median number of AZA cycles per patient was 4 (range, 2–64). The median age was 64.9 years (range, 25–88 years) and the median follow-up time for survival was 12.1 months. The 30-day mortality rate is 0%, and 60-day mortality rate is 4.9%. The favorable-, intermediate-, and adverse-risk subtypes included 4(6.6%), 29(47.5%) and 28(45.9%) patients, respectively. The significant differences in baseline characteristics between recipients administered AZA alone and those administered VEN-AZA were age (*P*=0.0160), ELN risk (*P*=0.023), underlying lung disease (*P*=0.023), treatment response (*P*<0.001) and neutropenia at initiation of AZA (*P*=0.032). The VEN-AZA cohort received more antifungal prophylaxis than the AZA-only cohort (*P*<0.001). Antifungal prophylaxis at the time of initiating therapy with VEN-AZA was posaconazole in 73.9% (n=17) and fluconazole in 4.4% (n=1).

**Table 1:** Baseline clinicopathological characteristics of AML patients treated with AZA only and VEN-AZA (n=61).

Characteristics	AZA only (N=38)		Ven-AZA (N=23)			*P*-value
	n or mean	(%) or SD	n or mean		(%) or SD	
Mean Age (years)	68.6	11.1	58.1		17.1	0.016*
AML subtype						
De novo	17	44.7	12		52.2	0.573
Secondary	21	55.3	11		47.8	
ELN risk						
Favorable	1	2.6	3		13.0	0.023*
Intermediate	20	52.6	9		39.1	
Adverse	17	44.7	11		47.8	
Comorbidity						
Lung disease	29	76.3	11		47.8	0.023*
Diabetes	12	31.6	4		17.4	0.222
Chronic kidney disease GFR<60mL/min	8	21.1	2		8.7	0.294
Liver cirrhosis	2	5.3	0		0.0	0.522
AZA administration						
Frontline	30	79.0	19		82.6	1.000
After prior AML treatment	8	21.1	4		17.4	
Treatment response						
CR	4	10.5	8		34.8	<0.001*
Cri	8	21.1	3		13.0	
PR	4	10.5	1		4.4	
SD	2	5.3	1		4.4	
PD	14	36.8	7		30.4	
MLFS	6	15.8	3		13.0	
Antifungal prophylaxis						
No	35	92.1	5		21.7	<0.001*
Fluconazole	1	2.6	1		4.4	
Posaconazole	2	5.3	17		73.9	
Neutropenia at initiation of AZA						
No	24	63.2	8		34.8	0.032*
Yes	14	36.8	15		65.2	
Thrombocytopenia at initial AZA						
No	21	55.3	12		52.2	0.815
Yes	17	44.7	11		47.8	
Prolonged neutropenia	6	15.8	6		26.1	0.342
IFI Event						
No or Possible	32	84.2	17		73.9	0.342
Probable or proven	6	15.8	6		26.1	

AML, Acute myeloid leukemia; AZA, Azacitidine; VEN, Venetoclax; SD, Standard deviation; ELN, European LeukemiaNet; GFR, Glomerular filtration rate; CR, Complete remission; CRi, Complete remission with incomplete hematologic recovery; PR, Partial remission; SD, Stable disease; PD, Progression disease; MLFS, Morphologic leukemia-free state; Prolonged neutropenia, Absolute neutrophil count (ANC) ≤500 cells/µL lasting >7 days; IFI, Invasive fungal infections.* P-value<0.05 was considered statistically significant

Patients who received VEN-AZA had a higher rate of neutropenia at initiation of treatment and prolonged neutropenia compared to patients administered AZA alone (65.2% vs 36.8%, *P*= 0.032, 26.1% vs 15.8%, *P*=0.342, respectively). IFI rates did not differ significantly between the VEN-AZA and AZA-only cohorts. (26.1% vs 15.8%, *P*=0.342).

### Patterns of infections

Amount 61 patients, there are 3 patients had Mycobacterium tuberculosis infection, 8 patients had Pneumocystis jirovecii infection, 20 patients had gram negative (GN) bacteremia(most are Escherichia coli), 12 patients had gram positive (GP) bacteremia(most are Staphylococcus aureus), and 6 patients had both GN and GP bacteria during AZA treatment. Amount patients had probable and proven IFI, there are 3 patients had PJP infection, 3 patients had GN bacteremia, and 2 patients had GP bacteremia during AZA treatment.

Amount patients had probable and proven IFI, there are 3 patients had PJP infection, 3 patients had GN bacteremia, and 2 patients had GP bacteremia during AZA treatment.Twenty-three cases of IFI (11 possible [18%], 11 probable [18%] and 1 proven [1.6%]) occurred during AZA-containing regimens. Thirty-eight patients (62.3%) had no evidence of IFI. All probable IFI cases were Aspergillus infections, most commonly involving the lung (11/12, 91.7%). Pulmonary IFI was diagnosed by microscopy of bronchoalveolar lavage (BAL) fluid or Aspergillus-positive serum by GM testing. One case of Fusarium spp. was classified as proven IFI because the skin culture was obtained under local anesthesia from sterile skin and soft tissue biopsy. This case occurred under posaconazole prophylaxis. Of those with probable/proven IFI (n=11), there are six patients under posaconazole prophylaxis, five patients change to voriconazole, and one patient change to Posaconazole. There are 5 patients didn’t receive antifungal prophylaxis, and four of them used echinocandin and one didn’t change the regiment. Of those with probable IFI, 7 patients received primary antifungal prophylaxis (1 received fluconazole and 6 received posaconazole). The clinicopathological characteristics of AML patients receiving AZA with and without antifungal prophylaxis are detailed in **Supplementary Table 1**.

Of the 38 patients on AZA alone, 6 (15.8%) had probable IFIs. Of the 23 patients administered VEN-AZA, 6 (26%) had probable or proven IFIs. The addition of venetoclax did not influence IFI incidence rates during AZA treatment. The majority of IFI cases (9/12; 75%) occurred during the first AZA cycle. Half of all IFI cases occurred in the context of neutropenia. In the 12 cases of IFI, four patients were alive at last follow-up. The cause of death in the remaining 8 patients was IFI (n = 7) and underlying malignancy (n = 1). **Table 2** details the IFI characteristics during AZA treatment.

**Table 2:** IFI characteristics during AZA alone or VEN-AZA treatment.

No.	Age, y/ Gender	AML subtype	ELN risk		AML setting	Treatment regiment	Bestresponse	No. of AZA cycles received at IFI diagnosis	IFI prophylaxis	IFI diagnosis	Clinical factor presents at IFI diagnosis (Site)	Microbiological factor presents at IFI diagnosis	Treatment outcome of IFI
1	65/M	sAML		Intermediate	Untreated	AZA	CR	12	Posaconazole	Probable	Lung	BAL GM (+)	Resolved
2	30/M	dnAML		Poor	r/r	AZA	CR	1	Fluconazole	Probable	Lung	serum GM (+)	IFI-RM
3	48/M	dnAML		Poor	r/r	AZA	CRi	1	Posaconazole	Probable	Lung	serum GM (+)	IFI-RM
4	48/F	dnAML		Intermediate	r/r	VEN-AZA	CRi	1	None	Probable	Lung	serum GM (+)	Death
5	64/F	dnAML		Poor	Untreated	AZA	CR	1	None	Probable	Lung	serum GM (+)	IFI-RM
6	69/M	sAML		Poor	Untreated	AZA	CRi	1	None	Probable	Lung	serum GM (+)	Resolved
7	81/M	sAML		poor	Untreated	AZA	refractory	1	None	Probable	Lung	serum GM (+)	IFI-RM
8	68/M	sAML		poor	Untreated	VEN-AZA	refractory	2	Posaconazole	Probable	Lung	BAL GM (+)	IFI-RM
9	36/F	dnAML		Good	Untreated	VEN-AZA	CR	1	Posaconazole	Probable	Lung	BAL GM (+)	Resolved
10	52/M	dnAML		Poor	Untreated	VEN-AZA	CR	1	Posaconazole	Proven	Skin/soft tissue	Fusarium spp.	Resolved
11	64/M	dnAML		Good	Untreated	VEN-AZA	CR	6	Posaconazole	Probable	Lung	serum GM (+)	IFI-RM
12	78/M	sAML		Poor	untreated	VEN-AZA	refractory	1	Posaconazole	Probable	Lung	serum GM (+)	IFI-RM

AML, Acute myeloid leukemia; AZA, Azacitidine; VEN, Venetoclax; ELN, European LeukemiaNet; IFI, invasive fungal infection; M, Male; F, female; dnAML, de novo AML; r/r, relapsed/refractory; CR, Complete remission; CRi, Complete remission with incomplete hematologic recovery; GM (+), galactomannan; IFI-RM; Invasive fungal infection-related mortality.

### Predictors for IFI

**Figure 2:**
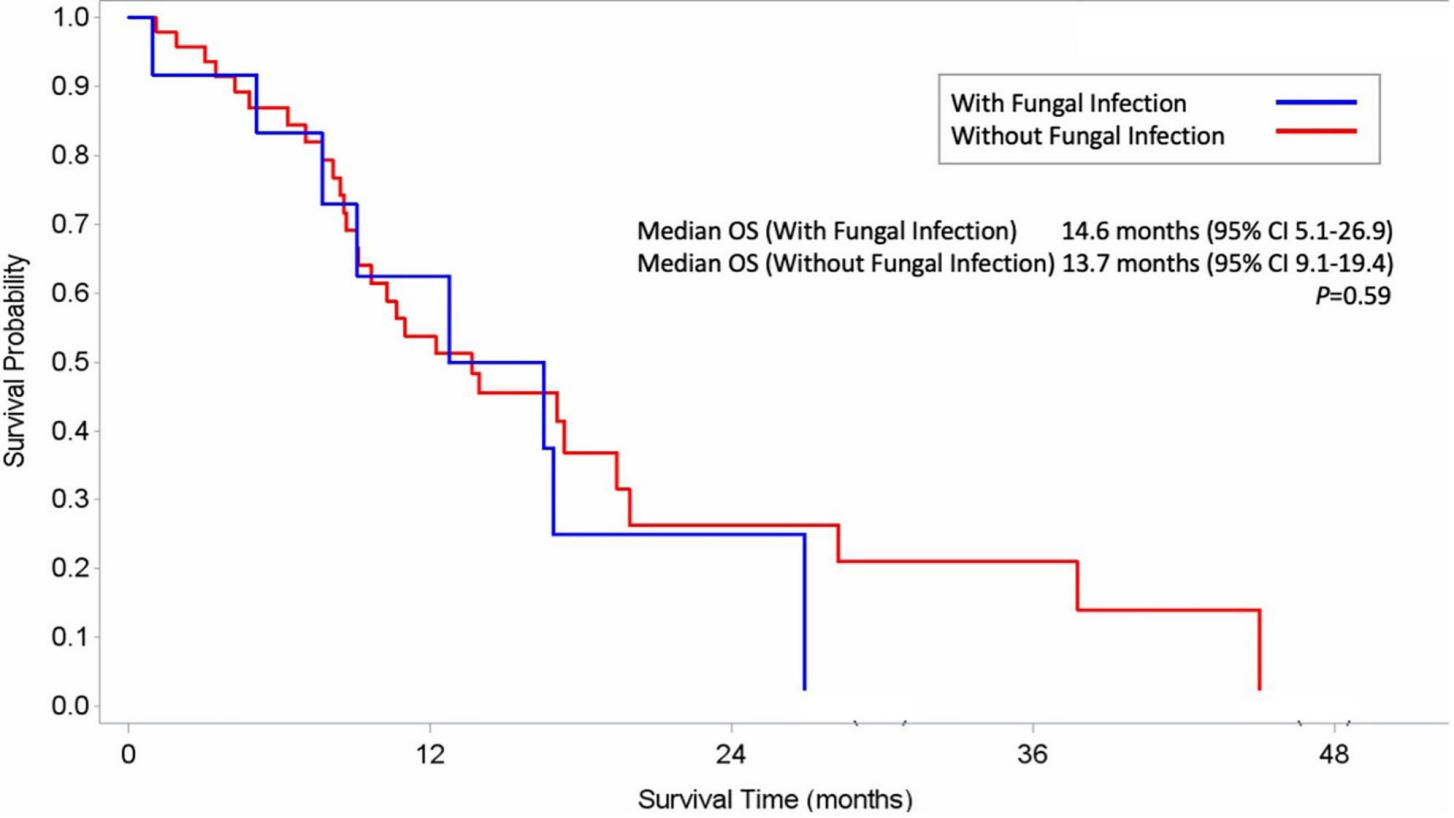
Kaplan–Meier Estimates of Overall Survival among AML patients with fungal infection **(A)** and without fungal infection **(B)**.

As shown in **Table 3**, an association was observed between the incidence of IFI and patient characteristics. The factors of patient age (*P*=0.667), *de novo* or secondary AML (*P*=0.518), treatment response (*P*=0.452) and neutropenia at initiation of AZA (*P*=0.597) failed to influence the risk of IFI during AZA therapy. Multivariate analysis confirmed that onset of probable/proven IFIs was significantly influenced by ELN risk classification (HR: 0.12, 95% CI: 0.02, 0.94, *P*=0.04) and prolonged neutropenia (HR: 5.66, 95% CI: 1.18, 27.15, *P*=0.03). As shown in **Supplementary Table 1**, a sensitivity analysis revealed a significant positive association between IFI and the variables of treatment response (*P*<0.001), antifungal prophylaxis (*P*=0.024) and prolonged neutropenia (*P*<0.001). In this study, median OS did not differ significantly between patients with and without IFIs during AZA-containing regimens (14.6 months vs 13.7 months; *P*=0.59) (**Figure 2**).

**Table 3:** Predictors of IFIs.

Variables	Univariate		Multivariate		
	HR (95% CI)	*P*-value	HR (95% CI)	*P*-value
Age (≤60 years/>61 years)		0.77 (0.24, 2.50)	0.667		
AML subtype (*de novo*/secondary)		0.68 (0.21, 2.17)	0.518		
ELN risk (favorable/non-favorable)		0.19 (0.04, 0.94)	0.042*	0.12 (0.02, 0.94)	0.044*
Comorbidities					
Lung disease (No/Yes)		1.46 (0.39, 5.39)	0.572		
Diabetes (No/Yes)		1.97 (0.51, 7.66)	0.328		
Chronic kidney disease (No/Yes)		0.92 (0.2, 4.27)	0.912		
Liver cirrhosis (No/Yes)		–	0.995		
AZA administration (Reference: front-line)					
After prior AML treatment		0.64 (0.17, 2.45)	0.519		
VEN-AZA (No/Yes)		1.99 (0.63, 6.36)	0.243	1.58 (0.36, 6.96)	0.544
Treatment response					
CR vs non-CR		0.64 (0.20, 2.04)	0.452		
Antifungal prophylaxis (No/Yes)		3.53 (1.01, 12.31)	0.048*	0.77 (0.13, 4.70)	0.777
Neutropenia at initial AZA (No/Yes)		1.36 (0.43, 4.32)	0.597		
Thrombocytopenia at initial AZA (No/Yes)		3.67 (1.08, 12.52)	0.038*	2.54 (0.60, 10.77)	0.206
Prolonged neutropenia (No/Yes)		4.71 (1.43, 15.51)	0.011*	5.66 (1.18, 27.15)	0.030*

HR, Hazard ratio; CI, Confidence interval; AML, Acute myeloid leukemia; ELN, European LeukemiaNet; AZA, Azacitidine; VEN, venetoclax; CR, Complete remission; Cox proportional regression; non-favorable: intermediated and adverse risk groups. * P-value<0.05 was considered statistically significant.

## Discussion

This retrospective assessment of IFI incidence rates among 61 patients with AML treated with AZA-containing regimens identified a markedly high IFI rate of 19.7%. Over half of the study population (52.5%) had secondary AML, and 20% of all patients received HMA therapy for relapsed/refractory disease. A non-favorable (intermediate- and adverse-risk) ELN risk classification and prolonged neutropenia were found to be independent predicator for IFIs. **Table 4** summarizes HMA regimens, antifungal prophylaxis, and IFI incidence rates in AML/MDS patients from selected published clinical trials and this study.

**Table 4:** A summary of HMA regimens, antifungal prophylaxis, and IFI incidence rates in AML/MDS patients from selected publications and this study.

Publications	Numbers of patients and diagnoses	HMA regimens	Antifungal prophylaxis	IFI incidence
Falantes et al., 2014	43 MDS pts 21 AML pts	AZA (≧2 cycles)	none	8/64 (12.5%) 6 *Aspergillus* spp. (probable)
Pomares et al., 2016	35 MDS pts 86 AML pts	AZA (≧1 cycle)	none	2/121 (1.6%) of all pts 2/49 (4.1%) pts with severe neutropenia 1 Aspergillus *fumigatus*. (probable); 1 *Candida albican* (proven)
Trubiano et al., 2017	49 MDS pts 19 AML pts	AZA (≧1 cycle)	29% posaconazole or voriconazole	8.8% (6/68) 5 *Aspergillus* spp. 2 Mixed growths 1 Mucormycosis
Ali et al., 2017	27 MDS pts 58 AML pts	DAC 10 days (≧3 cycles)	31% fluconazole	6/85(7%) 1 *Aspergillus* spp. (probable) 1 Mucormycosis (proven) 1 *Fusarium* spp. (proven)
Madry et al, 2019	150 MDS pts 59 AML pts 23 CMML pts	AZA (Not clarified)	52% fluconazole or posaconazole	14/232 (6%) 2 Aspergillus fumigates(proven) 10 Aspergillus spp. (probable) 2 *Candida tropicalis* (proven)
Kim et al., 2020	102 MDS pts 107 AML pts	AZA DAC (≧2 cycles of either drugs)	11% fluconazole	7/209 (3.3%) 4 *Aspergillus* spp. (probable) 1 *Scedosporium* spp.(proven) 1 *Fusarium* spp.(proven)
Latagliata R et al., 2020	234 MDS pts	AZA (Not clarified)	none	26/234 (11.1%) 13 Aspergillus spp. (probable)
Our study	61 AML pts	AZA (≧2 cycles)	34.4% posaconazole or fluconazole	12/61 (19.7%) 11 *Aspergillus* spp. (probable) 1 *Fusarium* spp. (proven)

HMAs, hypomethylating agents; IFI, Invasive fungal infection; AML, Acute myeloid leukemia; MDS, Myelodysplastic syndrome; pts, Patients; AZA, Azacitidine; DAC, decitabine; CMML, chronic myelomonocytic leukemia.

The incidence rates of IFIs in HMA-treated AML/MDS patients in previous studies range from 1.6% to 12.5% (Falantes et al., 2014; Pomares et al., 2016; Yang and Chen, 2017; Kim et al., 2020; Latagliata et al., 2020). However, these ranges need to be considered in the light of the fact that most reports on infections during AZA treatment include mixed patient populations with high-risk MDS and AML, different diagnostic workups, and different definitions for IFIs. Our analysis of a homogeneous cohort of AML patients undergoing AZA therapy confirmed a very high rate of IFIs (19.7%), with one-third of the patients receiving combination VEN-AZA treatment. A previous study recorded an IFI incidence rate of 10.3% in patients with AML (Kim et al., 2020).

In a Phase 1B study of VEN-HMAs administered to newly-diagnosed patients with AML, the IFI rate was 8%, which was attributed to the prophylactic use of echinocandin antifungal prophylaxis (DiNardo et al., 2019). One retrospective report describe a much higher incidence of IFIs in patients treated for relapsed or refractory AML compared with newly-diagnosed patients with AML (19% vs 5%; *P*=0.0498) (DiNardo et al., 2019). In our cohort, the majority of patients administered VEN-HMA regimens (19/23) had newly-diagnosed AML. The relatively high rate of IFIs (26%) in our study was reported in 6 of the 23 patients treated with VEN-AZA, 18 (78%) of whom received azole prophylaxis: among those 6 patients with IFIs, 5 (83%) developed IFIs during significant cytopenia and 1 of them received treatment for relapsed AML. The cause of prolonged neutropenia was considered to be the drug interaction between venetoclax and azoles, which are moderate-to-strong CYP3A4 inhibitors (Agarwal et al., 2017), and high serum venetoclax levels have been reported in Asian subjects (Cheung et al., 2018).

Based on evidence from a randomized phase III clinical trial, antifungal prophylaxis is strongly recommended for patients with AML or MDS who develop neutropenia after chemotherapy (Cornely et al., 2007). In that trial, posaconazole was statistically significantly superior to fluconazole or itraconazole, with proven and probable rates of IFIs of 2% and 8%, respectively (*P*<0.001). These data led to posaconazole becoming the first drug of choice for IFI prophylaxis (Cornely et al., 2007). A retrospective analysis of a clinical trial involving patients with AML and MDS treated with AZA has reported that the risk of IFI is very low per patient and per treatment cycle (1.6% and 0.21%, respectively) (Döhner et al., 2017). On the strength of these data, the European Conference on Infections in Leukaemia (ECIL) does not recommend antifungal prophylaxis in patients with low-to-intermediate risk MDS receiving HMAs (AZA or DAC) (Maertens et al., 2018). Due to the scant available data for AML patients who are elderly or have co-existing diseases, HMA regimens are deemed appropriate instead of standard chemotherapy for such patients (Tey et al., 2021). In our study, the choice of antifungal prophylaxis was at the discretion of the treating physician, according to the patient’s clinical condition. Interestingly, difference choices of antifungal prophylaxis did not appear to affect IFI rates. A higher proportion of neutropenic episodes were observed at initiation of VEN-AZA treatment compared with AZA alone (65.2% vs. 36.8%, *P*=0.032) and the rates of antifungal prophylaxis were higher in the VEN-AZA recipients versus the AZA-only cohort (78.3% vs 7.9%, *P*<0.001). Our study results are not enough on their own to prove the efficacy of posaconazole as antifungal prophylaxis, so further studies are warranted investigating other anti-mold agents in this setting. It is notable that in this study, the overall incidence of IFI rates in the no-prophylaxis group was 10% (4/40 patients), exceeding the 8% threshold for IFI incidence rates set by the European Conference on Infections for the recommendation of antifungal prophylaxis (Maertens et al., 2018).

Our data confirmed a significant higher incidence of IFIs in cycle 1 (9/12, 75%) and that prolonged neutropenia is a risk factor for IFIs. The IFI rate in patients with prolonged neutropenia was about 5 times higher than that in patients without neutropenia. Furthermore, more than half of the cases of IFI (7/12, 58%) were in patients who experienced prolonged neutropenia at during the first cycle. Therefore, anti-mold prophylaxis appears to be warranted during the early cycles of AZA treatment (cycles 1–2), given the higher incidence of IFIs during this period.

The ELN nonfavorable-risk subtype was significantly predictive of an increased incidence of IFI durng treatment with AZA; indeed, 27% of these patients developed one or more IFIs. The ELN risk classification has been shown to provide prognostic information in AML patients undergoing intensive chemotherapy as well as low-intensity regimens of HMAs (Döhner et al., 2017; Estey, 2020). Patients with an ELN nonfavorable-risk subtype have shown higher rates of induction-related mortality compared with favorable-risk patients (Keren-Froim et al., 2022). A high risk of induction-related death may be due to a high infection rate, including IFI. The high risk of IFI may be due to lack of an efficient immune response in AML patients with an ELN adverse-risk subtype. It is established that patients with AML present with immune dysfunction, characterized by T cell and natural killer cell function defects (Tang et al., 2020). It is also known that leukemia cells can induce immunosuppressive changes in other cells in the surrounding microenvironment, such as increases in the M2/M1 macrophage phenotype ratio, the number of myeloid-derived suppressor cell-like cells, and proportion of regulatory T cells (Brück et al., 2020).

Median OS did not differ significantly between patients with and without IFIs during AZA therapy (14.6 months vs 13.7 months; *P*=0.59) in our study. Different target groups (e.g., MDS, low-blast and high-blast AML counts) and treatment regimens may contribute to different treatment outcomes (e.g., OS) (Davoodzadeh Gholami et al., 2017; Kim et al., 2020). Our study included AML patients with low or high bone marrow blasts (20–30% and >30%, respectively) treated with AZA alone or combination VEN-AZA. Our study is limited by its retrospective setting, with data obtained from a single tertiary care cancer center and a small patient sample. Moreover, it is known that posaconazole prophylaxis can reduce the sensitivity of serum GM determination, but our institution did not use therapeutic drug monitoring for those patients who used posaconazole prophylaxis. A third of our population (31%) received anti-fungal prophylaxis, which may have resulted in some missed diagnoses due to a lack of satisfactory biological tests. In addition, our analysis excludes patients with possible mold infections, which may have led to an underestimation of the true incidence of infection. Another limitation is that the choice of antifungal agent during HMA therapy was decided upon by the attending physician’s decision, in response to the individual patients’ clinical conditions, so the durations of antifungal prophylaxis differed.

In conclusion, our study revealed d a high incidence of IFI in patients with AML treated with HMAs in Taiwan. Based on our results, the majority of IFI cases occurred during the early cycles of AZA (cycles 1–2). A nonfavorable ELN risk classification and the presence of prolong neutropenia were significant predictors of IFIs in our AML cohort. A combination of biomarkers and molecular testing with regular monitoring is necessary for early diagnosis of IFIs, such as serum/BAL GM assays, serum β-D-glucan measurements and an Aspergillus PCR. Prospective studies are required to determine the optimal choice of antifungal prophylaxis during AZA-containing regimens in AML patients.

## Data availability statement

The raw data supporting the conclusions of this article will be made available by the authors, without undue reservation.

## Ethics statement

The studies involving human participants were reviewed and approved by the Research Ethics Committee of China Medical University Hospital. The patients/participants provided their written informed consent to participate in this study. 

## Author contributions

The authors confirm their contribution to the paper as follows: study conception and design: S-TW, C-HC, and M-YL; data collection: S-TW; analysis and interpretation of results: T-TC, C-CL, and L-YB; draft manuscript preparation: S-PY, M-WH, and M-YL; wrote the manuscript: M-YL. All authors contributed to the article and approved the submitted version.

## Funding

This work was supported by grants from The Ministry of Science and Technology (MOST 111-2314-B-039-059) and China Medical University Hospital (DMR-112-010). 

## Acknowledgments

We would like to thank Iona J. M^ac^Donald from China Medical University for her editing of the manuscript.

## Conflict of interest

The authors declare that the research was conducted in the absence of any commercial or financial relationships that could be construed as a potential conflict of interest.

## Publisher’s note

All claims expressed in this article are solely those of the authors and do not necessarily represent those of their affiliated organizations, or those of the publisher, the editors and the reviewers. Any product that may be evaluated in this article, or claim that may be made by its manufacturer, is not guaranteed or endorsed by the publisher.

## Supplementary material

Supplementary Table 1 Univariate analysis of the risk factors for possible/proven/probable IFI in AML patients treated with Azacitidine (n=61)
