# Supplementary material for: High rate of invasive fungal infections during early cycles of azacitidine for patients with acute myeloid leukemia
Source: Front Cell Infect Microbiol. 2022 Nov 30;12:1012334. doi: 10.3389/fcimb.2022.1012334 (PMC9748082; doi:10.3389/fcimb.2022.1012334)
Supplement: Supplementary Table 1 — Univariate analysis of the risk factors for possible/proven/probable IFI in AML patients treated with Azacitidine (n=61) [file Table_1.docx]

**Supplementary Table 1. Univariate analysis of the risk factors for possible/proven/probable IFI in AML patients treated with Azacitidine (n=61)**

| Characteristics | No IFI  (n=38) | | Probable or Proven or Possible IFI (n=23) | | *P*-value |
| --- | --- | --- | --- | --- | --- |
|  | n or  mean | (%) or  SD | n or  mean | (%) or  SD |  |
| Mean Age (years) | 67.3 | 12.8 | 61.0 | 16.0 | 0.090 |
| Follow up time (months) | 11.8 | 10.6 | 10.6 | 7.3 | 0.633 |
| AML subtype |  |  |  |  |  |
| De novo | 17 | 44.7 | 12 | 52.2 | 0.573 |
| Secondary | 21 | 55.3 | 11 | 48.8 |  |
| ELN risk |  |  |  |  |  |
| Favorable | 2 | 5.3 | 2 | 8.7 | 0.327 |
| Intermediate | 21 | 55.3 | 8 | 34.8 |  |
| Adverse | 15 | 39.5 | 13 | 56.5 |  |
| Comorbidity |  |  |  |  |  |
| Lung disease | 24 | 63.2 | 16 | 69.6 | 0.610 |
| Diabetes | 10 | 26.3 | 6 | 26.1 | 0.984 |
| Chronic kidney disease, GFR<60mL/min | 6 | 15.8 | 4 | 17.4 | 1.000 |
| Liver cirrhosis | 2 | 5.3 | 0 | 0.0 | 0.522 |
| AZA administration |  |  |  |  |  |
| Frontline | 33 | 86.8 | 16 | 69.6 | 0.182 |
| After prior AML treatment | 5 | 13.2 | 7 | 30.4 |  |
| Novel agent combination |  |  |  |  |  |
| No | 26 | 68.4 | 12 | 52.2 | 0.205 |
| Venetoclax | 12 | 31.6 | 11 | 47.8 |  |
| Treatment response |  |  |  |  |  |
| CR | 7 | 18.42 | 5 | 21.74 | **<0.001*** |
| CRi | 6 | 15.79 | 5 | 21.74 |  |
| PR | 2 | 5.26 | 3 | 13.04 |  |
| SD | 1 | 2.63 | 2 | 8.70 |  |
| PD | 16 | 42.11 | 5 | 21.74 |  |
| MLFS | 6 | 15.79 | 3 | 13.04 |  |
| Antifungal prophylaxis |  |  |  |  |  |
| No | 29 | 76.3 | 11 | 47.8 | **0.024*** |
| Fluconazole | 0 | 0.00 | 2 | 8.7 |  |
| Posaconazole | 9 | 23.7 | 10 | 43.5 |  |
| Neutropenia at initiation of AZA |  |  |  |  |  |
| No | 21 | 55.3 | 11 | 47.8 | 0.573 |
| Yes | 17 | 44.7 | 12 | 52.2 |  |
| Thrombocytopenia at initial AZA, |  |  |  |  |  |
| No | 22 | 57.9 | 11 | 47.8 | 0.444 |
| Yes | 16 | 42.1 | 12 | 52.2 |  |
| Prolonged neutropenia | 0 | 0.0 | 12 | 52.2 | **<0.001*** |

**Abbreviations:** AML, Acute myeloid leukemia; AZA, Azacitidine; VEN, Venetoclax; SD, Standard deviation; ELN, European LeukemiaNet; GFR, Glomerular filtration rate; CR, Complete remission; CRi, Complete remission with incomplete hematologic recovery; PR, Partial remission; SD, Stable disease; PD, Progression disease; MLFS, Morphologic leukemia-free state; Prolonged neutropenia, Absolute neutrophil count (ANC) ≤500 cells/µL lasting >7 days.
